# N-of-1 Trials in Pediatric Oncology: From a Population-Based Approach to Personalized Medicine—A Review

**DOI:** 10.3390/cancers13215428

**Published:** 2021-10-29

**Authors:** Michal Kyr, Adam Svobodnik, Radka Stepanova, Renata Hejnova

**Affiliations:** 1 Department of Paediatric Oncology, University Hospital Brno and School of Medicine, Masaryk University, Cernopolni 9, 613 00 Brno, Czech Republic; 2 International Clinical Research Centre, St. Anne’s University Hospital Brno, Pekarska 53, 656 91 Brno, Czech Republic; 3 Department of Pharmacology, Faculty of Medicine, Masaryk University, Kamenice 5, 625 00 Brno, Czech Republic; adam.svobodnik@anova-cro.com (A.S.); radka.stepanova@anova-cro.com (R.S.) 222873@mail.muni.cz (R.H.)

**Keywords:** small samples, N-of-1, rare diseases, personalized treatment, pediatric oncology, design, statistical analysis

## Abstract

**Simple Summary:**

Clinical trials in pediatric oncology and personalized medicine are challenging due to the rarity of the disease, the low prevalence, and the ever-improving treatment outcomes. Many of the methods designed for small numbers and approaches used in classical population studies are not suitable for personalized pediatric oncology. There has been a change of perspective on the whole issue of rare diseases and personalized medicine. For example, a shift from a population to an individual perspective, generalizing from the individual to the population, using repeated measures and a within-subject design instead of parallel groups, exploring the variability instead of suppressing it, etc. N-of-1 should be understood as a whole range of approaches that fit the new inferential, evidential and analytical paradigms of modern medicine.

**Abstract:**

Pediatric oncology is a critical area where the more efficient development of new treatments is urgently needed. The speed of approval of new drugs is still limited by regulatory requirements and a lack of innovative designs appropriate for trials in children. Childhood cancers meet the criteria of rare diseases. Personalized medicine brings it even closer to the horizon of individual cases. Thus, not all the traditional research tools, such as large-scale RCTs, are always suitable or even applicable, mainly due to limited sample sizes. Small samples and traditional versus subject-specific evidence are both distinctive issues in personalized pediatric oncology. Modern analytical approaches and adaptations of the paradigms of evidence are warranted. We have reviewed innovative trial designs and analytical methods developed for small populations, together with individualized approaches, given their applicability to pediatric oncology. We discuss traditional population-based and individualized perspectives of inferences and evidence, and explain the possibilities of using various methods in pediatric personalized oncology. We find that specific derivatives of the original N-of-1 trial design adapted for pediatric personalized oncology may represent an optimal analytical tool for this area of medicine. We conclude that no particular N-of-1 strategy can provide a solution. Rather, a whole range of approaches is needed to satisfy the new inferential and analytical paradigms of modern medicine. We reveal a new view of cancer as continuum model and discuss the “evidence puzzle”.

## 1. Introduction

The treatment of children with cancer requires the integration of clinical expertise and external evidence [1]. Different sources of evidence are available for pediatric oncologists with regard to drug efficacy and safety. This ranges from individual expert opinions to the most valuable meta-analyses of randomized controlled clinical trials [2]. The traditional level of evidence pyramid [3] is the most frequently used tool for classifying sources of evidence according to their level of evidence by the Grading of Recommendations, Assessment, Development and Evaluation (GRADE) system [4,5].

One of the most important sources of evidence are population-based randomized clinical trials (RCTs) [5,6]. However, traditional RCTs cannot be used in all areas of pediatric personalized oncology for several reasons. One of the most important reasons is that cancer in children meets the criteria of a rare disease, and all methodological consequences related to small populations are thus applicable [7]. For patients coming from small populations, the design and analysis of RCTs faces many challenges [8,9,10,11]. It is difficult to optimize sample size and prove statistical clinical significance in an RCT in the context of pediatric oncology [12], and there are many specific requirements when conducting RCTs in children [13]. The relationship between disease prevalence and sample size in RCT has also been described [14]. Thus, new study designs or even fundamentally different approaches must be developed and applied in pediatric oncology. Limited progress has been made in the development of new treatments for children in oncology, necessitating the development of a new platform to speed up drug development [15].

## 2. Limitations of Traditional RCTs in Pediatric Personalized Oncology

In this section, we will discuss areas that are important for analysis and interpretation in evidence-based medicine. They are the most fundamental issues, reflecting different aspects of the whole evidence-based approach, but they are often forgotten. They form a central basis for our argumentation.

### 2.1. Heterogeneity and Average Treatment Effects

Simply put, in the traditional population-based approach to evidence, any treatment is considered effective if, on average, it is successful in the treated arm compared to the control. Such evidence is typically offered by well designed RCTs [16,17]. From the statistical perspective, inferences from conventional (e.g., parallel-group) RCTs are based on marginal models that are easy to implement and interpret. In various situations, they are the right tools to be used to answer important questions or address hypotheses. For example, it is legitimate to ask what the average cost of a specific treatment is, what the average difference is in the effects of a treatment between two groups of patients, or what the average proportions of patients who benefit from different treatments are. These are typical, correct, and legitimate investigational questions. However, these questions are asked by specific stakeholders, such as regulatory authorities.

However, a typical question a physician may ask is what treatment is effective for his/her patient. At this point, the physician usually makes an assessment for that individual based on evidence obtained from a study group to which the patient is thought to belong. Although in many cases the inference may be correct, the physician may encounter typical ecological fallacy [18] issues, such as Simpson’s paradox [19]. This means that conclusions that hold for a study population do not necessarily hold for an individual or a specific subgroup of the population. It typically arises when the treatment correlates with both intra-individual (subject-specific) and inter-individual (population-specific) variability, but in opposite directions for each, or when the outcome is dependent on another factor. For example, imagine treatment A is slightly better than B. The outcome is also highly dependent on gender, and the treatment is more effective in males than in females. As such, if the more effective treatment A is given preferentially to women, and thus has worse outcomes, we conclude that treatment A is worse, provided we do not know the association or cannot adjust for it in the model.

This complex issue, referred to as heterogeneity of treatment effects [20], is addressed in several papers [21,22]. It is another argument for reconsidering classical dogmatic evidence and its adaptation to pediatric personalized oncology, or in small populations and personalized medicine in general.

### 2.2. Traditional Population-Based vs. Personalized Subject-Specific View

In the personalized era of medicine, subject-specific inferences form the fundamental basis for decision-making [23]. They are derived from conditional models, which take into account either specific individual, time, or both, factors. The population vs. subject-specific approaches are addressed in the paper by Lindsey et al. [24]. Taking repeated measures for each individual represents an attractive option for increasing the power of a study or the effective sample size when populations are limited by either time, space, or both. In the personalized approach, additional value is given by the repeated measurement strategy. This enables inter-individual heterogeneity to be explicitly modeled [24]. Such information may be utilized, e.g., in models with random effects. Paying a small price in terms of power due to the increased uncertainty in these models, it enables the combination (as in basket trials or meta-analyses) of different diagnoses or groups of patients, practically increasing the sample size, and legitimately extending the inferences that may be drawn from the original study population. On the other hand, the value of a population-based perspective is not lost when conditional models are used. Marginal inferences may be derived from conditional models indirectly [24].

### 2.3. Sample Size and Variability

There are several limitations to the applicability of traditional population-based RCTs in pediatric oncology. The most important limitation is related to sample size. In conventional phase II–III trials, usually, several hundreds or thousands of patients are enrolled. The number of patients that must be enrolled in a particular trial is assessed through the process of power analysis based on frequentist theory. The number of patients required to prove the expected clinical effect statistically significant is calculated. As for other rare diseases, the European Medicines Agency (EMA) guideline on clinical trials in small populations is applicable for trials in pediatric oncology [2]. From a regulatory perspective, no special statistical techniques applicable for small populations, which cannot be used in large populations, exist. In pediatric clinical trials, recruitment may be more difficult in comparison to trials in adults [25]. If the study does not have enough power to detect a difference, it is not ethical to start the trial.

The sample size needed for a clinical trial is determined by several factors. Among the most important are the expected effect size and variability. Small-sample-size clinical trials are more prone to variability and thus are only able to prove the significance of large clinical effects [26]. The higher the variability, the more patients need to be enrolled. An important source of variability is the heterogeneity of the disease itself [27].

From the statistical perspective, it is important to reduce the so-called “bio-noise” as one of the sources of variability. One way to deal with variability is to increase the sample size. If we consider bio-noise as “the sum of avoidable and unavoidable non-systematic errors in the design and conduct of a trial” [2], then if we wish to minimize the sample size, it is necessary to reduce all the “bio-noise”. A good example of bio-nose in clinical trials is low-quality (poor accuracy, bias, etc.) or completely missing data due to loss to follow-up [2]. Improving study design, e.g., obtaining good-quality data, avoiding drop-outs by making the study more patient-friendly with higher patient compliance rates, using continuous measures rather than categories, etc., can reduce some of the bio-noise as well as reducing sample size.

Until recently, different sources of variability have not been considered in clinical trials. As stated in the EMA guideline, “Variability (whether in terms of disease phenotype, underlying pathophysiology, pharmacodynamics or pharmacokinetics) is a threat to successful drug development. Efficient study design and analysis requires as clear an understanding as possible of all of these potential sources of variability.” [2] the biological variability is perceived as a threat rather than offering potentially valuable information. On the contrary, in the era of personalized medicine, it is crucial to distinguish between variability that carries information, i.e., biological variability, and noise, which is a non-systematic error. In population-based (parallel-group) trials, individual treatment effects cannot be estimated [21]. Both types of variability are thus treated the same as a threat that masks differences between study treatments and need to be statistically handled through power analysis. If just a small and unknown group of patients is to profit from a new treatment under evaluation, only a small effect is predicted for the whole study population, which results in the need to appropriately increase the number of patients. In this situation, the number that must be treated as the cost of observing an effect in a single patient (number needed to treat) increases significantly. In smaller populations, sufficient power may not be achieved in the sample size, but can be realized by decreasing the variability by targeting more homogeneous populations. The process may be carried out by utilizing more restrictive inclusion/exclusion criteria, enrichment and biomarker-guided designs, or various paired/matched settings. Unfortunately, these techniques not only compromise the external validity of a trial [20,21,28], but they also do not address the crucial fact that in personalized medicine, variability offers information that should be utilized rather than combated.

**Figure 1:**
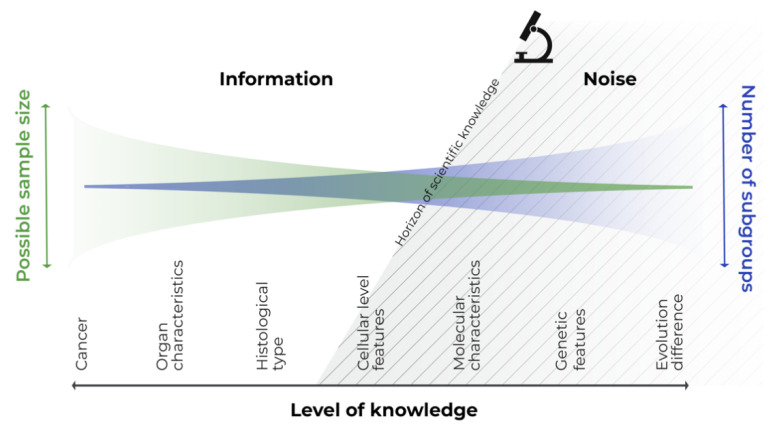
Continuous scale disease model.

We need to realize that there is no bounded, homogeneous subgroup of patients or diseases. Let us consider cancer, which we have been battling for a long time [29], from the perspective of all cancer patients with a single general disease. If we focus in more detail, we assess the different types and affected organs, histopathological and cellular pictures, and identify specific molecular or genetic subgroups. Finally, we can identify the individual patient with the disease, at a specific site and time, and even identify subparts of the tumor itself [30]. All these intersections only represent snapshots of the biological continuum of the disease–patient unit, which reflect knowledge about the disease at that specific time. Consider any section of this continuous scale disease model; the variability to the left of this section is known information, and that to the right is not yet known information, or information that cannot be utilized or captured by the study design (see Figure 1). The left-hand side information can be utilized in a statistical model as a covariate, whereas the right-hand side information can be handled only as noise.

In personalized medicine, disease is considered on an individual level, and this permits us to move into the right-hand side of the model, as technological advancements offer us greater information about the disease. We can see, that modern personalized approaches involving the incorporation of molecular features into clinical trials are possible for many childhood cancers [31]. The trials are still able to achieve power, study several cohorts, and demonstrate benefit, especially in more common cancers such as leukemia, CNS tumors, neuroblastomas, etc. On the other hand, if modern personalized approaches are also to be followed in very rare entities or subgroups, it complicates the performing of population-based analyses to extract evidence, given the decreasing sample size of the target study populations [32]. Perhaps we should rather make use of personalized procedures based on individual cases, pooling the evidence and then translating the inferences to a broader target population.

### 2.4. Randomization

Randomization is an integral procedure in RCTs. It ensures unbiased estimates by balancing samples between the study arms, with respect to the known and unknown covariates. However, in small clinical trials, it is difficult to control bias through standard randomization procedures [8,33]. It has been reported that a sample size of >200 patients is required to balance the samples for a given covariate [34]. Thus, in rare diseases, even if randomization is feasible in principle for such small populations (of several tens), one of the most important effects of randomization is reduced.

### 2.5. Recruitment

Patients who are not willing to participate in any trial may further limit the already small numbers available. Performing clinical trials and willingness to participate in a pediatric study, in general, can be a problem, especially in young adults [35], under certain circumstances, with different diseases, socioeconomic factors [25], etc. On the one hand, recruitment rate may be low if the potential benefit of the study for the patient is low, e.g., in prevention, screening, side effects, or quality of life issues [35], and concerns about risk, including the risk of the control treatment, intervention and testing burden, etc., arise. It is known that patients are more willing to participate in alternative designs, such as cross-over, N-of-1, delayed start randomization, or similar techniques, in comparison to traditional RCTs, as they have the option to test all the treatments [36]. On the other hand, recruitment levels are known to be high for pediatric oncology trials where clinical practice and clinical research has almost converged [25].

### 2.6. Patient Horizon

This is another issue arising in rare diseases, and therefore in personalized pediatric oncology. The so-called “patient horizon” [37] is a *future target population likely to benefit from a trial result*. To clarify this concept, let us take two extreme hypothetical situations. In the first example, all patients from the target population, including future patients, are randomized in a 1:1 ratio to the effective and ineffective treatment arms, respectively. In the second situation, no trial is performed at all. In the first case, half of the patients in the trial, and hence in the whole target population, receive an ineffective treatment as a price for knowing the relative treatment efficacies of the two treatments [38]. The same chances of a benefit are derived by the patient, i.e., 50/50, from such a trial as would have been obtained if the trial had not been conducted at all (thus we would have learned nothing about the efficacy of the treatment), and the treatment had been administered randomly based on the physician’s guess. Let us term such a situation a “trial benefit paradox”. An optimal trial size balances both extremes, i.e., the trial population and a future target population, and maximizes the number of patients who benefit. In large trials on common diseases, the patient horizon can be considered infinite, and almost no trial size will compromise the benefit derived by the population. However, in small populations with extremely rare diseases, the above paradox arises. The exact number of patients in a trial may not be known, but the order of magnitude of the optimal number for a simple two-arm trial design can be calculated using *the square root of the size of the patient horizon under consideration* [37]. For example, for a finite population—taking into account both the prevalence of the disease and the life cycle of the new treatment—of 1000 subjects, the optimal trial size is a few dozen. Considering disease rarity, especially in the era of molecular medicine, and the life cycle of newly developed drugs, the issue of the target population size (the patient horizon) becomes relevant not just in pediatric personalized oncology, but in medicine in general.

### 2.7. Control Arm

One of the drawbacks of traditional RCTs is the problem of the control treatment arm, regarding whether a placebo could be used and what is the best control treatment [25]. The issue of the control arm can be briefly summarized in the following comparison. An effective/better treatment compared to a placebo/significantly inferior treatment should yield a large treatment effect, hence the need for a smaller sample size. However, this will make patients less willing to participate because they are at risk of receiving such a control/inferior treatment. Current best standard treatment, not a placebo, is required by the Declaration of Helsinki [25]. From this perspective, sample size is a matter of balancing effect size, ethics, and willingness to participate by defining the control arm.

Both of these extremes may arise in pediatric oncology—rapidly developing areas with highly effective treatments, or, on the other hand, aggressive or incurable diseases. In the first case, the statistical problem is the ever-decreasing effect size, while in the second case, it is the risk of inferior treatment.

### 2.8. Recruitment Rates and Outcome Measures

Paradoxically, the problem of the rarity of pediatric cancer is further accentuated by improvements in treatment efficacy and survival. This means that fewer events, i.e., smaller effective sample sizes, are available for analysis over time. Moreover, outcome measures tend to become less reliable. Popular and robust measures, such as overall survival, are being replaced by alternatives, e.g., EFS, PFS, size of tumor shrinkage, tumor markers, etc. They are either easier to use in practice, have statistically higher power, but may be less reliable surrogates, have a different meaning, or in combination [39,40]. The general need to collect sufficient data in a reasonable timeframe has led to the formation of large international consortia, such as COG, SIOP, etc. [41,42]. Such international collaboration overcomes to some extent the problem of small samples or slow recruitment rates, and allows analysis on the scale of a large population. The availability of patient-level data from collaborative groups represents another advantage. They may be used in secondary multivariate analyses, and give rise to stratification parameters that are validated in subsequent trials. Unsurprisingly, again, the size of the resulting stratified groups is reduced. We can surmise that this collaborative approach alone will not remain sufficient given the continuous improvements treatment results.

## 3. Analytical Approaches for Small Populations and Their Applicability in Pediatric Oncology

The question of the applicability of traditional population-based RCTs for rare diseases has been addressed in recent years by a number of studies [6,15,18,19,43,44,45] and guidelines [2,46]. Several general principles can be identified on which the required sample size depends. We summarize them in Table 1.

There are several general methods that address the sample size and effectiveness of traditional RCTs. These methods include sequential design, n-adjustable design, sample size re-estimation during interim analyses, multi-arm trials, factorial design, and pre–post-trial design [47]. They reduce the total number of patients or the total duration of the trial phases, similarly to the principle of quantity discounting. This means that trials utilizing the above methods work more efficiently, either reducing the number of subjects, time, or both, but require the usual number of patients as RCTs. We think, that these designs do not address the inherent problem of the small number of rare diseases, and are not appropriate for them.

One useful, rather statistical approach is changing the operating characteristics of the trial, i.e., relaxing type I, II, or both, errors, or targeting larger effects [32]. This is a common technique used in various oncology consortia. Renfro et al. [32] reviewed this issue well, among others. In their overview, they showed that performing smaller trials with relaxed operating characteristics more frequently offers greater survival benefits in the context of low-incidence diseases than insisting on low error rates in large trials. In other words, with less certainty about the results of each individual study, patients do better in the long run. This finding is also consistent with the theory of patient horizon previously described. This should encourage researchers and clinicians to utilize this approach of “making small and uncertain but more frequent steps towards the goal”. Minimal therapeutic costs may be represented by different values of type I and II errors depending on different disease burden; the traditional type I error of 5% may be too aggressive (too high) for non-life-threatening conditions, whereas it is too conservative for deadly diseases and optimal type I error would be much higher in the latter case [48].

One design that can be considered despite various difficulties is the cross-over design [49,50]. This is a sort of paired parallel group design with within-subject swapping of the treatments being evaluated. It uses repeated measures and makes within-subject comparisons, which provides greater power and reduces patient numbers by controlling for inter-individual variability. It is usually suitable for drugs with a short-term effect on symptoms, and which do not permanently change the course of the disease [49]. It is usually given for chronic, non-lethal conditions, such as hypertension, pain, asthma, etc. The wash-out period should also be considered so that the trial can be properly designed to avoid a potential carryover effect.

Another option for small populations is adaptive designs. There are a number of adaptive approaches available that have been reviewed by Chow and Chang [51]. Generally speaking, an adaptive design is a pre-planned feature of a trial that modifies a further experimental condition to make the trial efficient, usually by means of changing the selection/grouping of “winners” out of several parallel arms, changing the tested hypotheses, or increasing a specific population (enrichment design) based on the results of the previous (learning) phases of the trial, to make the later (confirmatory) phases more efficient, etc. Particularly in adaptive designs, it is crucial to ensure both the internal (rendering unbiased treatment effects) and external (allowing generalization to a large population) validity and integrity (meaning robust risk–benefit balance and ethical design) of the trial [52,53]. A systematic review of the adaptive designs in pediatric clinical trials has been performed [54].

One of more popular designs is an adaptive randomization design that allows the future modification of treatment assignment based on previous treatment assignments [55]. One may balance randomization between the study arms with respect to covariates or treatments (covariate-adaptive and restricted randomization, respectively), or the objective may be to maximize treatment efficacy and minimize failures during the trial (play-the-winner, so-called response-adaptive design). Because the adaptation is based on treatment response in the latter case, it is not suitable for a trial with long time-to-response or treatment duration, as it may delay the trial’s completion.

Integrative data analysis [56] and interrupted time series analysis [57] are, we think, interesting and promising options for small samples in personalized oncology. The first case involves the statistical analysis of a single data set consisting of multiple separate samples, e.g., from different existing studies, that have been pooled into one. The potential advantages are increased statistical power, increased sample heterogeneity, and increased frequencies of low base-rate cases. The latter two factors would normally be considered disadvantages in, for example, classical meta-analyses of population trials. However, in the context of personalized medicine, they should be seen as valuable information. The second case is one type of N-of-1 study, in which the change in the variable of interest over a period of time is assessed. In a classical time-series analysis, a continuous variable is needed, which may represent a major problem in pediatric oncology. On the other hand, randomization is not an integral part of this method, as each subject serves as his/her own control. These techniques are therefore of particular interest for pediatric personalized oncology, wherein only single cases or small series may be available, and randomization may become an ethical issue [38]. Evidence from pooled and observational data or registries becomes more relevant for similar techniques. An example of such a combination of approaches can be found in our recent paper [58], in which the aggregation of heterogeneous data, and time series analyses of survival data, were performed.

Complex work has been done within the ASTERIX project [47] on small samples in general. However, some of the approaches see similar issues arising in pediatric oncology as were described above. A delayed start randomization design [47] enables the creation of a kind of paired or crossed design, enabling all patients to eventually receive the treatment. However, this is suitable for a disease with a slow, constant progressive course, which is not the case for the rapidly progressing high-risk pediatric cancers in MTD-based regimens. Adaptive survival trials with sample size reassessment [59] handle only “wasted” time, and subjects on a trial or a sequence of trials, based on interim analyses. They do not seem to be suitable for pediatric personalized oncology, where just a few patients, or even a single one, may be the target population horizon.

Another option for small samples is a multi-arm group sequential design [60], which combines multi-arm and sequential approaches based on interim analyses. It utilizes available patients more efficiently, saving up to 20% of the sample size depending on the design. However, it was originally developed for normally distributed endpoints or other conferrable types (i.e., binary) with asymptotic normality, making it suitable only for certain situations with a number of patients usual for classical trials. It is a technique that reduces numbers and time, but more effectively than both techniques separately. It may be interesting to apply it for classical treatment based on fewer individuals, e.g., risk-based stratifications, where sufficient numbers are expected.

Dynamic borrowing using power priors that control type I error [47] is another interesting option. In rare diseases, and in either pediatric cancers, personalized oncology, or both, it is helpful to use previous information (the prior) from historical studies. Such power priors are expressed as a parameter that, in most situations, directly translates into a fraction of the sample size of the historical study that is included in the analysis of the new study. However, the possibility of borrowing data from a historical trial will usually be associated with an inflation of the type I error [47]. Therefore, Nikolakopoulos [61] suggested a new, simple method for estimating the power parameter in the power prior formulation, which is suitable when only one historical dataset is available. This method is based on predictive distributions, and is parameterized in such a way that the type I error can be controlled by calibrating the degree of similarity between the new and historical data.

Similarly, previous information may be employed in Bayesian methods [47]. Bayesian statistics use probability distributions, often including the probability of belief in the intervention before the start of the trial (the prior). For normally distributed outcomes, an assumption of the variance needs to be made to inform the sample size needed, which is usually based on limited prior information, especially in small populations. When using a Bayesian approach, the aggregation of prior information on variance with newly collected data is more formalized. The uncertainty surrounding prior estimates can be modeled with prior distributions. The authors adapted the previously suggested methodology to facilitate sample size re-estimation. In addition, they suggested the employment of power priors in order to control the operational characteristics. We think that correctly utilized historical information is one of the most important approaches to be used in the analysis of rare diseases. Controlling the overall classical or Bayesian operational characteristics is crucial.

The last two approaches mentioned above represent the use of extrapolative evidence. Not only historical data but, e.g., data from adults or different sub-populations can be used as extrapolative evidence [62,63]. There is also a framework suggested by the EMA [64].

A very interesting option proposed by Hoff [65] is using the progression-free survival (PFS) ratio as the primary endpoint, which is the ratio of PFS on the last treatment to the PFS on a new, e.g., targeted, treatment. It is a type of paired design developed for survival data, eliminating the between-patient variability. Although the correlation between the paired failure times plays an important role [66], we think that it is an interesting option, especially for addressing the issues of small samples and personalized oncology.

It is also important to consider different phases of drug development and the absolute frequencies of each rare entity. Leukemias, CNS tumors, neuroblastomas, etc., although rare in the absolute measure, they comprise the most frequent pediatric cancers. Conventional trial approaches through international collaborative groups, e.g., in the leukemia trial (ClinicalTrials.gov Identifier: NCT03643276) or in brain tumors [67], will still be the best standard of evidence. On the other hand, risk stratification will still result in small numbers in sub-groups [32].

Most of the methods may be considered for both new and already marketed drugs tested in the repurposed use. In the latter case, the prior information can be used in an extrapolative way.

## 4. N-of-1 Trials in Pediatric Oncology

### 4.1. General Aspects of N-of-1 Trials

N-of-1 trials are studies wherein the effects of different treatment options are tested in a cross-over setting within one patient [68]. The first documented experiments of such a design in humans are from 1676 [69]. However, the whole concept was introduced for the first time in the early 1980s [70].

In 2011, the Oxford Centre for Evidence-Based Medicine classified N-of-1 trials as Level 1 evidence useful for treatment decision-making in individual patients [71]. However, N-of-1 trials are not used very frequently by investigators; the main reasons for this are summarized by Kravitz et al. [72]. A review of published N-of-1 trials between 1985 and 2013 was published by Li et al., and a total of 112 such trials were identified in the medical literature from this period [73,74]. N-of-1 trials could be combined with traditional RCTs, and such meta-analyses are also possible [75].

The advantages of N-of-1 trials over traditional RCTs have been well described [76]. The main goal of conducting N-of-1 trials is to counter the problem of the difficulty in the generalization of results from traditional RCTs, and the inability to apply them to individual patients [77]. The use of collective experience to generate expectations for an individual is an example of reference class forecasting [78]. The aggregation of N-of-1 trials for the evaluation of new interventions is considered as an alternative study design to traditional RCTs for such situations when the recruitment of patients is a challenge [36]. Sometimes, N-of-1 trials are automatically considered as randomized, double-blind and multiple crossover comparisons of interventions and control treatment [36].

Another problem of traditional RCTs is that patients with comorbidities are not usually enrolled, and thus the results are difficult to interpret in clinical practice settings [79]. It has been well documented that N-of-1 trials are more acceptable for patients, their parents, and investigators, mainly because of the possibility of deriving an investigational product and not only using a control treatment (sometimes placebo) following randomization in traditional parallel-group RCTs [80].

N-of-1 trials could be more easily performed nowadays because of the prevalence of electronic wireless monitoring devices [81].

### 4.2. Applicability of N-of-1 Trials in Pediatric Oncology

N-of-1 trials have been used mainly in psychology [82]. Because of the cross-over design, these studies are most suitable for the evaluation of treatments with rapid effect onset, and for diseases in which patients quickly return to a stable baseline after treatment, without any significant carryover effect [83]. This is why N-of-1 studies have so far mainly been used for chronic diseases, such as arthritis, fibromyalgia, chronic airflow limitation, gastric reflux, hypertension, and others [68,74,84]. Regarding future use, the recommended therapeutic areas for N-of-1 trials include diabetes, glaucoma, hyperlipidemia, and asthma [78].

Although N-of-1 trials are very well accepted by physicians, patients, and parents in pediatric research [72], their application in pediatric oncology is rare [74]. Kravitz [85] classified N-of-1 trials into three main categories: (1) routine clinical care, (2) N-of-1 clinical service, and (3) N-of-1 trials conducted as research. Other possible classifications of N-of-1 trials include (1) a single N-of-1 trial and (2) a planned series of multiple N-of-1 trials [85].

For the following discussion of the applicability of N-of-1 studies in pediatric oncology, we have divided them into two categories: (1) N-of-1 trials as a tool to find the best treatment for the individual patient without any ambition to generalize the outcomes, and (2) N-of-1 trials performed to generalize the results for populations.

### 4.3. N-of-1 Trial to Find the Best Treatment for the Currently Treated Patient

The motivation for conducting this type of N-of-1 trial is to find the best treatment for the current individual patient [83]. Thus, it has a patient-centered objective. Many unpublished N-of-1 trials of this type have been performed in routine clinical practice, and they are usually focused on the assessment of already approved treatments [68]. In a non-randomized setting, it can also be considered the routine practice carried out by every physician when evaluating any given treatment or a dose of a drug. The possible conclusions of such a practice are either maintaining the treatment when effective, or modifying (changing or adding a drug, or modifying the dose) if ineffective or presenting with excessive toxicity.

### 4.4. N-of-1 as a Research Tool

The N-of-1 approach has a population-centered objective. The objective of this type of N-of-1 trial is to estimate the treatment effect in a whole population [68]. N-of-1 trials carried out to estimate a population treatment effect have already been applied in pediatric oncology [86]. The difference between this and the previous type of N-of-1 trial is that the benefit for an individual patient is secondary [85]. In order to obtain population-level estimates, individual trials are combined, and aggregated data are evaluated using various techniques [36,68]. Random effect models or Bayesian hierarchical models seem to be most appropriate. There are also guidelines for reporting single-subject studies, such as the single-case experimental design (SCED) for behavioral sciences [87], or a CONSORT extension for N-of-1 studies (CENT) in medical sciences [88,89]. Different kinds of N-of-1 trials are possible, such as controlled and randomized, or observational, based on comparative effectiveness research methods [83]. To expand our understanding of how to generate population-based evidence, one could consider this approach as a meta-analysis, in which the individual trials are the individual cases to be aggregated. In conventional meta-analysis, fixed effects are the favored and easiest to understand approach for pooled estimates. Such estimates may be used only for inferences regarding those populations used in that meta-analysis. This is often useful because, in population-based trials, inferences are made for the unknown part of the target population that is defined within the trials. However, in personalized medicine and single-patient trials, each patient is a different target population. It is necessary to be able to extend the interpretation beyond the patients under evaluation to the yet unknown target populations. Therefore, models with random effects are much more suitable for this purpose. We recently used the aggregation and random effects approach on observational survival data to demonstrate the benefit of individualized treatment in children with high-risk solid tumors [58].

Several requirements for classical N-of-1 trial design must be met. Most of them can rarely be achieved in pediatric oncology. These aspects are outlined in Table 2, together with their possible applicability.

As is obvious from Table 2, we can summarize that only a few application strategies for N-of-1 trials are feasible in pediatric oncology. These strategies include the aggregation of multiple patients to compensate for the impossibility of a sufficient number of repeated measurements in patients with aggressive diseases with MTD-based chemotherapy, using classical N-of-1s in which either low-dose metronomic or long-term targeted treatment is used for individualized strategies, and the individual setting of design parameters with respect to assumptions, feasibility, and cost/benefit.

From a certain point of view, the utilization of the N-of-1 design in pediatric oncology to generate data for the regulatory approval of new treatments is the most challenging application. According to the EMA [2], N-of-1 trials are an acceptable source of data for regulatory purposes in small populations; however, these trials have many of the same limitations as crossover trials. N-of-1 trials were initially intended to be a part of the early phase of drug development [90].

**Figure 2:**
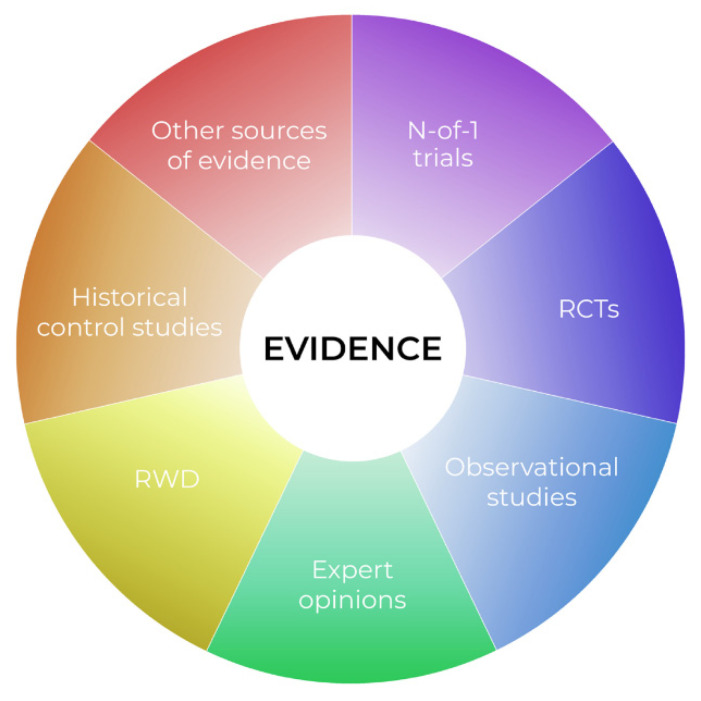
The complementary puzzle of evidence in rare diseases.

We dare say that classical population-based trial goals are unachievable in very rare diseases, and in personalized medicine in particular. Indeed, if we move again to the right side of Figure 1, and approach the horizon of personalization, we find that individual cases are available, instead of large samples. At this point, applying classical methods for large populations, if at all possible, more or less compromises the external validity of any such trial. It is also obvious that in pediatric oncology, utilizing such a specific design as classical N-of-1 also has limitations. We suggest that the evidence pyramid rather be reshaped in the “complementary puzzle of evidence” (Figure 2) for rare diseases and personalized medicine. Such an idea can be sensed from the EMA statement “In very rare diseases, the combined evaluation of single case studies may be the only way to provide evidence. Also combined analysis of individual case reports or observational studies should be considered.” [91]. The idea is that the source of evidence is dependent on the individual situation, such as data availability, the treatment under consideration, or various individual patient factors, with no one source of evidence being universally significantly more important than the others. Thus, in personalized medicine, not only treatment, but also evidence sources should be evaluated individually. For example, a small series of case reports on a very rare disease with treatment aimed at a well-defined target, with known biological processes and a large, obvious treatment effect, may represent relevant and sufficient evidence. In another case, a Bayesian approach extrapolating prior knowledge, e.g., from adults to a pediatric population with a lower incidence of disease, for a drug acting on the same target, may be the optimal tool to increase the study’s power and provide evidence. A classical N-of-1 trial offers a suitable tool for the long-term targeted treatment of an indolent disease with a valid surrogate that can be easily monitored.

Our aim is not to provide guidance on what evidence is suitable in what situation. Rather, we want to introduce the evidence puzzle idea, wherein classical principles of hierarchy of evidence and trial phases are inapplicable. Physicians usually compose similar puzzles of evidence in routine clinical practice when considering the off-label use of a treatment. However, it is an extremely difficult task for regulatory authorities seeking to apply objective and general rules. We can imagine that each source of evidence can be further classified as either trusted/relevant or possibly biased, as a quality measure. How to combine all these factors is, however, not obvious. Further research is needed so we can offer general guidance on this issue.

### 4.5. Economic Consideration N-of-1 Trials

It has been shown in non-oncological chronic diseases that N-of-1 management of patients saves on the costs of expensive drugs [92,93]. Of course, cost-effectiveness depends on a number of factors. In oncology, the biologicals that are repeatedly being developed are costly, and biosimilars may not be an effective cheaper alternative [94]. Since, on the other hand, genetic biomarker diagnostic tools become increasingly available with lower costs per gene tested, there is clear potential for savings and improved cost-effectiveness attached to utilizing the N-of-1 management of patients, even when using these expensive drugs.

## 5. Conclusions

In small samples and personalized pediatric oncology, numerous factors limit the utilization of classical “high-level” populational evidence. There is a clear trend in the field of rare diseases and personalized medicine. We are moving from parallel groups to repeated measures and within-subject designs, exploiting interindividual variability rather than fighting it, drawing conclusions for groups from individual patients, and using evidence of varying significance.

Various statistical techniques may be employed to overcome some of the issues and improve the utilization of available data. Utilizing conditional models that allow inference from patient to population better suits personalized medicine. Using surrogates or combined endpoint measures may improve the statistical power. Relaxing operating the characteristics of trials allows for more frequent studies, and is, surprisingly, more beneficial in the long run. Aggregation, combining, and series analysis compensate for the lack of repeatability of measurements in a single subject, and increase sample size. The use of mixed models and random effects that explicitly handle inter- and intra-individual variability while protecting the hierarchical structure from aggregate patterns will allow extrapolation beyond the study population. Bayesian statistics increase power by utilizing previous or translational data, and offer different hypotheses and an alternative statistical philosophy. Specific derivatives of classical N-of-1s, such as paired comparisons enriched by aggregations, may be useful in personalized pediatric oncology. The evidence should be individually evaluated in personalized medicine.

In personalized pediatric oncology, no single type of N-of-1 study will provide the solution. Rather, the N-of-1 strategy should be considered as a whole conceptual approach and a philosophy of evidence collection. Different existing approaches may be appropriately adapted, combined, and utilized in such an N-of-1 conceptual framework at different phases of drug development.

## Figures and Tables

**Table 1:** General principles affecting sample size.

Principle to Be Addressed	Examples
Affecting overall trial efficacy	factorial, multi-arm, sequential designs, adaptive, enrichment designs
Changing operational characteristics	inflating type I/II errors, targeting larger effects
Handling interindividual variability	Cross-over, paired designs, time series, PFS ratios
Utilizing efficacy of endpoint measure	Surrogate continuous measures, EFS
Trial power and sample size enrichment	Integrative analysis, aggregation/combining, Bayesian approaches, meta-analyses, dynamic borrowing and power priors, historical controls

**Table 2:** Assumptions of N-of-1 trials (as research) and their limitations in pediatric oncology.

N-of-1 Trials’ Assumptions	Rationale for the Assumption	Limitation in Pediatric Oncology	Possible Solution for Pediatric Oncology
The half-life of the medication being tested is short [36]	Rapid washout as cycles alternate	Half-life itself is not a limiting factor	Most drugs either in supportive care or cancer treatment can be tested
Carryover effect (the effect of the treatment stops soon after it is discontinued) [76]	Either slow-onset, carryover effects, or both, can compromise the validity of the trial [83]	Limiting factor. Mainly for targeted treatment with drugs of which the effect is not well known. Classical MTD-based chemotherapy cycles are usually designed to begin after the wearing off of the acute toxicity effect. Although treatment effect may be proportional to the toxicity, it is assumed to last longer, thus carrying over *.	Could be addressed via model design using reversed switches. Hardly utilized for cancer treatment efficacy. More suitable for the supportive part of treatment.
There is rapid onset/offset of the biological action of the medication [36]	Slow onset can compromise the validity of the trial [83]	Not limiting for classical MTD-based chemotherapy with assumed relatively rapid effect. Maybe a limiting factor for metronomic or targeted treatment where a slower effect is assumed.	Targeted treatment is given to patients based on life expectancy to incorporate the assumed latency of treatment effect. Used for more indolent courses of disease, where a longer period for evaluation may be designed. It is a relative measure with respect to the biological behavior of the disease.
The effect of the medication can be measured using a validated outcome measure [36]	Choosing the right scale, a key factor in ensuring that real benefits and burdens (net clinical effect) are being measured	Very limiting factor for most oncological patients using survival models with usually one or just a few events. Usual measure is a non-event (stable disease or continuing remission), which cannot be measured until that event occurs	Using valid surrogate measure (a tumor marker) where possible, usually measured on a continuous scale. Aggregating similar subjects to generate pseudo repeated measures (when addressed, e.g., in hierarchical models).
Treatments causing permanent or only slowly reversible effects not possible (e.g., surgery) [83]	If the underlying condition is altered, the results as the trial progresses for the individual will not be interpretable	The crucial limiting factor mainly for classical chemotherapy. There is usually one or very few opportunities to combat cancer with a more aggressive course for further progression or relapses. Evolutional character and resistance development is similarly limiting for targeted treatments	Use for more indolent diseases or supportive treatment. Use of aggregating approach for aggressive disease.
The medication or condition is important enough to warrant an N-of-1 trial [36]	N-of-1 trials are intensive for the participant and resource-intensive for the participating unit	Assumption is fulfilled	
Chronicity and stability of the disease	To reduce the chance that differences between parts of each trial cycle are caused by the treatment vs. comparator and not changes in the underlying condition (not the effect of the medication) [36]	Crucial limiting factor. High-grade cancers in children limit treatment opportunities	Suitable for indolent cancers on metronomic or long-term targeted treatment
Fixed number of cycles needed [36]	In typical N-of-1 trials the minimum number of cycles is three cycles, more cycles, better statistical power [36]	Crucial limiting factor for high-grade cancers. Reluctance to switch treatment, which is obviously effective.	Using aggregated approaches. Suitable for diseases with indolent courses on metronomic treatment. Use on treatment with an uncertainty of effects
Randomization	Obtain treatment effects unbiased for uncontrollable covariates	Is possible in principle, it is an ethical issue, limiting its randomization within subject due to the limited number of opportunities	Randomize between subjects, use aggregate approaches
Blinding	To reduce bias due to placebo or related effects	Not relevant, thus not limiting since treatment effect in oncology is hardly dependent on placebo or “confounder” factor	Trials are usually designed as unblinded

* In MTD-based chemotherapy, the treatment/toxicity effect should not be attributed to the time of administration of a drug, but to the whole period of its biological effect that was just triggered by the drug.
